# Swimming through sand: connectivity of aquatic fauna in deserts

**DOI:** 10.1002/ece3.1741

**Published:** 2015-10-23

**Authors:** Ashley L. Murphy, Alexandra Pavlova, Ross Thompson, Jenny Davis, Paul Sunnucks

**Affiliations:** ^1^ School of Biological Sciences Monash University Melbourne Victoria 3800 Australia; ^2^ Institute for Applied Ecology University of Canberra Canberra Australian Capital Territory 2617 Australia

**Keywords:** Arid, dispersal, drylands, ephemeral, freshwater, gene flow, intermittent, river, spring, stream

## Abstract

Freshwater ecosystems in arid regions range from highly fragmented to highly connected, and connectivity has been assumed to be a major factor in the persistence of aquatic biota in arid environments. This review sought to synthesize existing research on genetic estimation of population connectivity in desert freshwaters, identify knowledge gaps, and set priorities for future studies of connectivity in these environments. From an extensive literature search, we synthesized the approaches applied, systems studied, and conclusions about connectivity reached in population genetic research concerning desert freshwater connectivity globally. We restrict our scope to obligate aquatic fauna that disperse largely via freshwaters and exclude those with active aerial dispersal abilities. We examined 92 papers, comprising 133 studies, published from 1987 to 2014. Most described studies of fishes and invertebrates in the deserts of Australia and North America. Connectivity declined with increasing scale, but did not differ significantly among arid regions or taxonomic classes. There were significant differences in connectivity patterns between species with different dispersal abilities, and between spring and riverine habitats at local scales. Population connectivity in desert freshwaters is typically most influenced by the ecology of the species concerned and hydrological connectivity. Most studies did not assess predefined models of connectivity, but described gene flow and/or genetic structure. Climate change and anthropogenic impacts worldwide are likely to increase the incidence and impact of habitat fragmentation in already threatened desert freshwaters. To reduce this risk, biodiversity conservation and environmental management must address connectivity, but often the required information does not exist. Researchers can provide this by explicitly considering the effects of hydrology and species’ ecology on connectivity, and incorporating these into connectivity models, which are vital for understanding connectivity in desert freshwaters.

## Introduction

**Figure 1:**
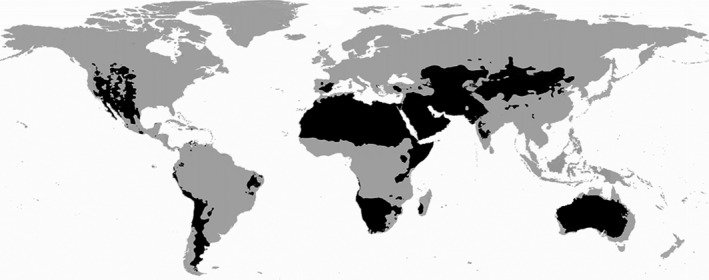
Deserts of the world (black), based on Köppen–Geiger climate data (adapted from Peel et al. 2007).

Arid and semi‐arid regions, here referred to as deserts, cover more than 30% of the world's surface area (Peel et al. 2007). They dominate the Australian and African continents, and significant portions of Asia, North America, and South America (Fig. 1). Deserts are defined by an annual rainfall of no more than 500 mm and an annual evaporation rate equivalent to 95% or more of this total (Meigs 1953). These environments are among the most inhospitable places on Earth, but almost all contain aquatic habitats. Despite these habitats being typically restricted in number and extent, they are important for many desert species. Desert freshwaters include springs, river networks, lakes, and pools that may be ground‐ or surface‐water fed. These range across a continuum of temporal permanence, with many classified as temporary (Kingsford 2006).

Desert freshwater ecosystems provide vital resources for a wide range of taxa as well as valuable ecosystem services for local people (Kingsford 2006). They provide habitats for aquatic biota, including invertebrates, fishes, amphibians, and turtles, and can act as ecological or evolutionary refugia (Davis et al. 2013). Despite their importance, these freshwaters and their inhabitants are less well studied than those in mesic environments (Sada et al. 2005; Kingsford 2006; Box et al. 2008; Vazquez‐Dominguez et al. 2009). They are also among the world's most threatened biomes, with water extraction, habitat degradation and flow modification directly impacting biodiversity and ecosystem functions (Abell 2002; Dudgeon et al. 2006; Palmer et al. 2008; Vorosmarty et al. 2010). These threats are intensified further by the natural isolation of freshwater habitats (Bates et al. 2008; Davis et al. 2013) and global climate change (Woodward et al. 2010; Jaeger et al. 2014). Together, these threats are expected to further increase the fragmentation of desert freshwaters.

In the face of these threats, effective biodiversity conservation and environmental management is required, and understanding how populations are spatially and temporally connected is imperative for predicting management outcomes (Hermoso et al. 2011; Hughes et al. 2013). Persistence in fragmented desert freshwaters, which are typically spatially and temporally variable, often requires that species maintain wide geographic ranges to enable dispersal when hydrological connectivity allows (i.e., during floods). Such connectivity is also spatially and temporally variable (Meffe and Vrijenhoek 1988). Unless noted otherwise, we use the term connectivity to refer to population connectivity – the combination of genetic connectivity, determined by the effects of gene flow on evolutionary processes within populations, and demographic connectivity, defined by the contribution of dispersers from one population to the growth rate of another (Lowe and Allendorf 2010).

Species’ ecology, physical connections between habitats provided by environmental factors (structural connectivity), and their interactions are the drivers of desert freshwater connectivity (Hughes et al. 2013). Relevant species’ ecology encompasses many biological factors, including dispersal ability, physiological tolerance, niche breadth, and reproductive potential (Öckinger et al. 2010). These factors greatly affect how a species is distributed, under what conditions it can persist, and how its distribution can change. Many desert‐dwelling freshwater species are highly tolerant of environmental extremes, such as high temperature, salinity (e.g., Glover 1971; McNeil et al. 2011), and many have excellent dispersal abilities (e.g., Stanley et al. 1994; Unmack 2001; Fagan et al. 2002). Such characteristics may also allow them to take advantage of typically limited structural connectivity.

**Figure 2:**
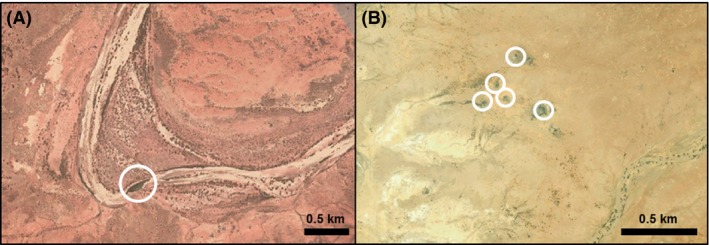
Examples of structurally disconnected freshwater habitats (circled) in arid central Australia: (A) a disconnected waterhole on the Finke River (24.58°S 133.30°E) and (B) disconnected spring outflows in the Hawker Spring complex (28.41°S 136.18°E). Source: Google Earth.

In freshwater ecosystems, geomorphology and hydrology are the most important environmental factors determining structural connectivity. Geomorphologically, rivers and most other aquatic habitats in desert regions do not differ significantly from those in wetter regions (Nanson et al. 2002). However, hydrologically, dryland freshwaters are far more variable than those in mesic areas, and often experience long periods without flows, leading to disconnection (Fig. 2) and reduced structural connectivity (Carini and Hughes 2006). There are also differences in hydrology between deserts. For example, rivers in Australian deserts are almost entirely reliant on a rainfall regime that is among the most variable and unpredictable in the world (Van Etten 2009). In contrast, many North American desert freshwaters are fed by more seasonal inputs, including snowmelt, meaning that temporary flows are somewhat predictable (e.g., Bogan and Lytle 2007).

Here, we conduct a review of studies of connectivity of obligate aquatic species in arid regions worldwide, to inform future research and conservation of desert freshwater biodiversity. Our aim is to provide the first synthesis of methodologies and results in this field, using an increasingly common and appreciated quantitative approach that allows strong conclusions to be made from a diverse literature (e.g., Beheregaray 2008; Storfer et al. 2010). We begin by reviewing the connectivity models that can be utilized in desert freshwater connectivity research, and then integrate knowledge of desert freshwater connectivity across regions, scales, habitat types, taxonomic classes, and dispersal abilities. We restrict our review to taxa that generally require freshwaters for dispersal and exclude species with active aerial dispersal mechanisms. We focus exclusively on studies that use molecular data, as genetic estimates of population history offer an increasingly cost‐effective approach to understanding past and present connectivity that cannot be practically performed any other way for many locations and organisms.

## Connectivity Models for Desert Freshwaters

Connectivity models (also referred to as population structure or genetic structure models) are utilized in many connectivity studies and have been extensively used in freshwater research. Wright's (1943) Panmixia and Isolation by Distance were the first connectivity models. The former proposes that a species is able to disperse easily across its entire distribution, meaning its populations are highly connected and genetically homogenous (see Table 1 for details of the population genetic structure of each model). In contrast, Isolation by Distance (IBD) proposes a decrease in genetic similarity with geographic distance throughout the distribution of a species, reflecting dispersal limitation. IBD is common in nature and may be more appropriate as a null hypothesis in connectivity studies than is panmixia, depending on the spatial scale of study relative to the scale on which dispersal occurs (e.g., Amos et al. 2014).

**Table 1:** Models of Desert Freshwater Connectivity (adapted from “Models of Genetic Structure”, Hughes et al. 2013)

Model	Population genetics description	References
Panmixia	No genetic structure among populations, extensive gene flow	Wright (1943)
Isolation by Distance	Genetic structure between populations strongly correlated with geographic distance	Wright (1943)
Isolation by Resistance	Genetic structure between populations strongly correlated with resistance distance (a measure of gene flow likelihood between two locations)	McRae (2006)
Isolation by Environment	Genetic structure between populations correlated with environmental heterogeneity and not geographic distance	Wang and Bradburd (2014)
Stream Hierarchy Model	Genetic structure between populations strongly correlated with the physical structure of the stream network	Meffe and Vrijenhoek (1988)
Headwater Model	Genetic structure between populations of headwater specialists strongly correlated with geographic distances in headwaters	Finn et al. (2007)
Death Valley Model	Strong genetic structure between populations resulting from loss of connectivity, no contemporary gene flow	Meffe and Vrijenhoek (1988)

Owing to the hierarchical nature of many aquatic habitats and the spatially and temporally disconnected flows in many deserts, specialized connectivity models have been developed for desert freshwaters. Meffe and Vrijenhoek (1988) proposed two models to depict connectivity in North American desert freshwaters, the Stream Hierarchy and Death Valley models. The Stream Hierarchy Model states that patterns of connectivity should follow the dendritic patterns of the stream network, incorporating both geographic distance and habitat connectivity (Hopken et al. 2013; Hughes et al. 2013). In contrast, the Death Valley Model assumes extremely low connectivity between sites and high, spatially unstructured differentiation among populations and thus no relationship between geographic and genetic distance.

A small number of specialized connectivity models extended these early ones and can be applied to desert freshwaters. These include the Headwater Model, which applies principally to headwater taxa, and assumes the presence of temporary aquatic connections between catchment boundaries or some terrestrial dispersal ability. The Headwater Model predicts that species will be able to utilize connectivity between headwaters in adjacent streams, not necessarily in the same catchment, and that these populations will be more genetically similar than those in streams with nonadjacent headwaters (Finn et al. 2007; Hughes et al. 2009).

Recent approaches have expanded connectivity studies to include variables other than geographic distances. Isolation by Resistance utilizes spatially explicit predictive surfaces of connectivity, with landscape resistances to dispersal conditioned on landscape features, which can be compared with predictions from the above models (McRae 2006). A range of landscape variables, in many combinations, can be used to calculate resistance distances (e.g., Cañedo‐Argüelles et al. 2015; Morán‐Ordóñez et al. 2015). In contrast, Isolation by Environment offers a framework for examining the effects of ecological and environmental heterogeneity on connectivity, while controlling for the effect of geographic distance (Wang and Summers 2010; Wang and Bradburd 2014; Morán‐Ordóñez et al. 2015). The expected pattern is one where genetic differentiation increases with environmental differentiation, independent of geographic distance, and is generated by natural or sexual selection against immigrants, reduced hybrid fitness or biased dispersal (Wang and Bradburd 2014). Finally, a number of process‐based approaches to test Isolation by Environment have been built (Wang and Bradburd 2014). These include Isolation by Adaptation (Nosil et al. 2008) and Isolation by Ecology (Claremont et al. 2011; Shafer and Wolf 2013), but neither have yet been applied to desert freshwaters.

## Methods

A dataset of empirical studies was analyzed to give an overview of the trends in desert freshwater connectivity research, including methodologies, study systems, and results. The dataset was compiled by searching all databases of the Web of Science^®^ collection on 2 March 2015. The search terms were (“genetic*” OR “connectivity” OR “population structure”) AND (“freshwater*” OR “river*” OR “stream*” OR “spring*”) AND (“desert*” OR “arid*” OR “dryland” OR “rangeland” OR “temporary” OR “ephemeral” OR “intermittent” OR “fragment*”). To remove results from unrelated fields of study, searches were restricted to five biological categories (Environmental Sciences, Ecology, Evolutionary Biology, Marine & Freshwater Biology, Zoology), and the journal *Conservation Genetics*. Results were refined to include only “articles” (papers).

For inclusion, studies had to analyze population structure or connectivity, using molecular methods, in locations defined as “deserts” as above. Taxa were restricted to obligate aquatic fauna, defined as animals that spend all or most of their life history in freshwater. To restrict our review to taxa that require freshwaters for their dispersal, species with active terrestrial or aerial dispersal mechanisms were excluded, as were those that disperse via marine waters (at least within the studied system). Species that can disperse passively, either aerially or through water, for example via phoresy, wind, or in‐stream drift, were included. Because of the specialized ecology and connectivity of species living in underground waters (stygofauna), studies of these taxa were excluded.

A total of 3171 papers were found in the search, of which 70 met all inclusion criteria. The reference lists of included papers were consulted, and 22 relevant papers were added, resulting in a final dataset of 92 papers (for details and references of all included papers see Appendix S1 in Supporting Information). Several studies examined the same species at the same sites; in these cases, only the most recent study was included. To confirm the effectiveness of our search terms, we consulted the database of Australian freshwater connectivity studies compiled by Hughes et al. (2013): our search criteria found 13 of the 14 relevant articles included therein.

Publication details were obtained from each paper for analyses of temporal trends. Genetic marker/s and analytical methodologies used to infer or test connectivity or gene flow were recorded to gauge what analyses were possible and the power of inferences (see Table 2 for definitions of different methodologies). Connectivity models named and/or tested were also recorded (see Table 1).

**Table 2:** Descriptions of study variables recorded and tested for effect on connectivity and the categories within

Variable	Description
Analytical Methodology	The methodology used to estimate gene flow
Deterministic	Deterministic methods included inferences of population structure based on *F*_ST_ or other genetic distance measures, and nested clade phylogeographic analyses (Templeton 1998)
Probabilistic	Probabilistic model methods included approximate Bayesian computation (ABC; Beaumont 2010), coalescent approaches that estimate levels of gene flow [e.g., IMa (Hey and Nielsen 2004, 2007) and MIGRATE (Beerli 2006); reviewed in Kuhner 2009)], and assignment methods (assignment tests, genetic mixture analyses and parentage analyses; reviewed in Manel et al. 2005)
Habitat	The habitat type in which the study was conducted
River	Connected surface‐fed systems
Pool	Disconnected surface‐fed systems
Spring	Groundwater‐fed systems
Multiple	A combination of two or more of the above habitat types
Scale	The hydrological scale at which the study was conducted (note that some studies were conducted at multiple scales)
Within‐System	Within a river catchment, or pool or spring complex, local scale, that is, with freshwater hydrological connections
Between‐Systems	Between river catchments, or pool or spring complexes, within the same basin, that is, with possible freshwater hydrological connections
Between‐Basins	Between rivers, pool or spring systems, within different basins, that is, with no freshwater hydrological connections
Dispersal Ability	The perceived dispersal ability of the species studied, based on descriptions of dispersal in the reviewed papers (not genetic patterns) or where dispersal ability was not described, based on species’ biology or that of related species
Low	Species with maximum likely dispersal not exceeding the local, within‐system scale, for example, weak‐swimming fish, some mollusks
Moderate	Species with maximum likely dispersal not exceeding the between‐system scale, for example, strong‐swimming fish, invertebrates with drifting larval stage
High	Species with maximum likely dispersal at the between‐basin scale, for example, taxa with passive aerial or terrestrial dispersal abilities

Where multiple species were included within a paper, each taxon was treated as an additional study, with a final dataset including 133 studies. To check for under‐studied topics and compare patterns of connectivity, the following characteristics of each study were recorded: taxonomic class, study region/s, spatial extent (maximum straight‐line distance between any two sampling sites), scale, habitat, and species’ dispersal ability (classifications of the latter three as per Table 2). Red List status was recorded for vertebrates, the only included group to have been largely evaluated by the IUCN (2014).

The connectivity models concluded as best fit were recorded for each study to compare the conclusions among different paper approaches and study parameters outlined above. Where different conclusions were reached for different locations or scales within a study, these were recorded as additional conclusions. As many studies considered multiple scales, conclusions were recorded for each scale, giving a total of 141 conclusions. Where papers did not explicitly provide a connectivity model, we categorized their conclusions as no, restricted, or high gene flow, based on their descriptions of gene flow or genetic structure.

For statistical analysis, the conclusions made in each study about connectivity (description of gene flow or connectivity model) were grouped into three categories: high connectivity (which included high gene flow and panmixia), restricted connectivity (including restricted gene flow, Isolation by Distance and the Stream Hierarchy model), and no connectivity (no gene flow and the Death Valley model). The prevalence of these categories was compared among the following variables recorded for each study: analytical methodology, scale, region, habitat, taxonomic class, and dispersal ability. To test whether the conclusions reached differed with these variables, we used Pearson's chi‐square contingency test in R (R Core Team 2014). Significance of temporal trends of methodologies was tested by calculating Pearson's correlation coefficient in R.

## Results

### Publications

Relevant papers included in our review were periodically published from 1987 until the early 2000s; publication rates increased through to 2010, and declined post‐2010. The majority of papers were published by lead authors in the USA (47%) and Australia (29%), with the rest from Portugal (10%) and ten other countries, including Brazil, Chile, and eight European countries (15%). Papers were published in 30 different journals, most in discipline‐specific journals, the most common being *Molecular Ecology* (21%), *Freshwater Biology* (14%), and *Conservation Genetics* (12%).

### Methodologies

A total of eight molecular marker classes were used to assess connectivity. The most common marker was mitochondrial DNA (mtDNA; 66% of papers), followed by microsatellites (36%), allozymes (22%), nuclear DNA sequences (nDNA; 9%), and amplified fragment length polymorphisms (AFLP; 8%). The remaining classes, restricted fragment length polymorphisms (RFLP), randomly amplified polymorphic DNA (RAPD), and single‐primer amplification reaction (SPAR), were each used in fewer than 4% of papers. Most papers (55%) utilized one class of marker, 41% used two classes, and 3% used three classes.

There was a strong temporal component to which genetic marker classes were applied. Allozymes were the only markers used until 1996. From 2001, mtDNA and microsatellites became the main markers, with RFLP, AFLP, and RAPD used mostly (but rarely) from 2001 to 2006. Studies utilizing nDNA sequences first appeared in 2010. The number of marker classes used showed, at most, a small increase over time (*r* ^2^ = 0.04, *P *=* *0.05), while the number of individual loci used showed no significant trend (*r* ^2^ = 0.03, *P *=* *0.10). Almost 19% of papers used just one locus, in most cases mtDNA. At the other end of the scale, 15% of papers used 15 or more loci, generally allozymes.

Analytical methods fell into two main groups – 60% of papers estimated gene flow using deterministic models, including *F*_ST_, other genetic distances or genetic structure (nested clade phylogeographic analyses was included in this category), while 40% used probabilistic models, including coalescence, approximate Bayesian computation, and/or assignment methods. The analytical methodology used showed a strong change over time. The proportion of papers using deterministic models showed a significant decline (*r* ^2^ = 0.52, *P *<* *0.001). In contrast, probabilistic models showed a significant increase in usage (*r* ^2^ = 0.52, *P *<* *0.001) from 2003 and have been used in more studies since 2011.

Most studies did not consider a range of connectivity hypotheses, and Isolation by Distance and Panmixia were almost always the only ones explicitly tested. The Stream Hierarchy and Death Valley models were the next most common models tested (18% and 3% of all studies, respectively), while Isolation by Resistance was tested in just two studies (2%). There were no studies testing the Headwater Model, Isolation by Environment, or the other “Isolation by” models in the included papers, although the Headwater model was tested in one superseded paper (Finn et al. 2007). Overall, 25% of papers identified a best‐fit connectivity model for their system, while the rest described only the degree of gene flow (51%) or genetic structure (24%). The proportion of papers testing connectivity models showed no significant change over time (*r* ^2^ = 0.07, *P *=* *0.23). Eighteen percent of papers based on deterministic models concluded a connectivity model, compared with 35% of those based on probabilistic models.

### Study systems

**Figure 3:**
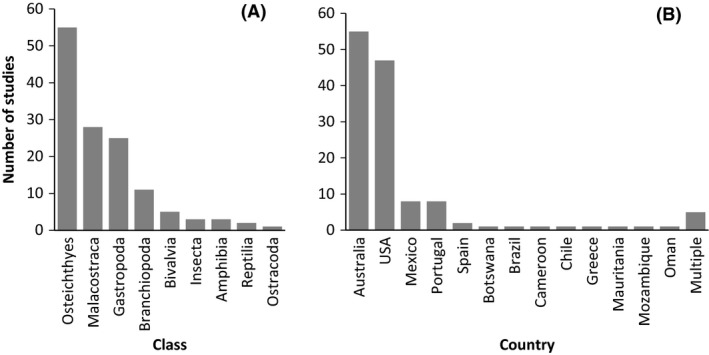
Number of studies examined of connectivity in desert freshwaters according to (A) class of study taxa and (B) country of study.

Of the 92 papers, 77% examined just one taxon, 12% two taxa, and 11% studied three or more taxa to a maximum of six. Overall, 107 species were included, of which 45% were vertebrates (fish, amphibians, and reptiles). Of the vertebrates, 46% were threatened species, 28% non‐threatened, and the rest unevaluated (IUCN, 2014). The 133 included studies incorporated nine taxonomic classes, although five of these (Bivalvia, Ostracoda, Insecta, Amphibia, and Reptilia) together accounted for just 10.5% of studies (Fig. 3A). Species with high dispersal abilities (11% of studies) were less studied than those with moderate (42%) or low (47%) dispersal abilities.

The vast majority of studies were restricted to one of twelve countries (Fig. 3B), with just 4% of studies crossing international borders. Studies were almost always conducted in developed countries, principally the USA and Australia, with smaller numbers in Europe. The studies incorporated eight global arid regions, but 94% of studies were conducted in just three – North American deserts (42% of studies), the Australian arid zone (41%), and Mediterranean Basin (11%). The spatial extent of studies ranged from 1 to 3500 km (mean 484 km). Most studies were performed within riverine (54%) or spring (34%) habitats, with a small number in pools (5%) and multiple habitats (7%).

Most studies considered several scales; 19% considered all three (see Table 2 for scale descriptions) 41% considered two (mostly the two smallest scales, 67%); and 40% considered only one (predominantly, 75%, the within‐system scale). Overall, 80% of studies examined connectivity at the within‐system scale, 61% at the between‐systems scale, and 37% at the between‐basins scale.

### Connectivity patterns

**Figure 4:**
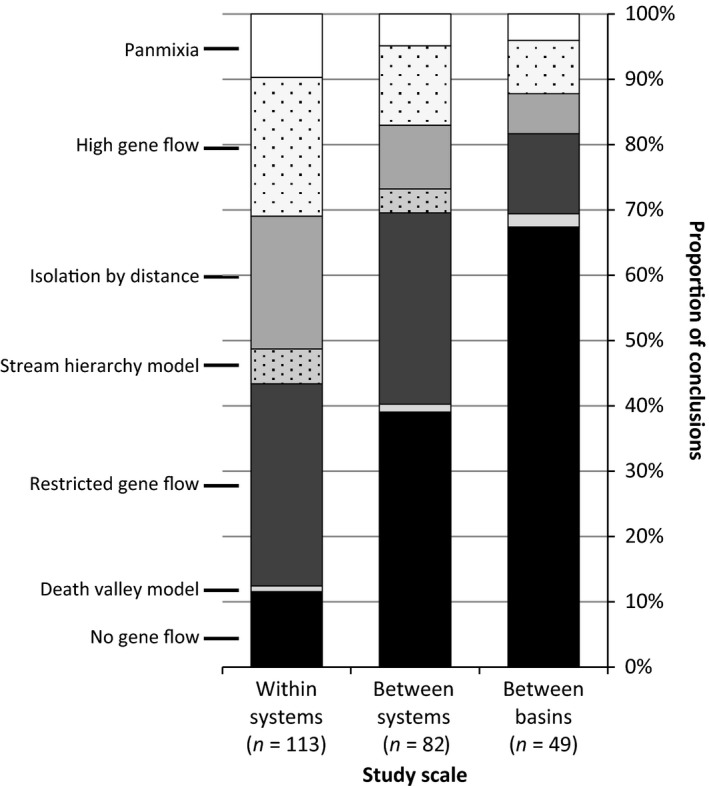
Percentage of studies of desert freshwater taxa that invoked each of seven connectivity models or gene flow descriptions, at three scales.

A clear pattern of decreasing connectivity at larger spatial scales was apparent (Fig. 4). When all conclusions about connectivity were combined into three categories (high, restricted, none), connectivity was found to differ significantly between the three scales (*χ* ^2^ = 53.63, df = 4, *P *<* *0.0001). The number of systems with no connectivity increased from 12% at the within‐system scale to 69% at the between‐basin scale, while the number with high and restricted connectivity decreased as spatial scale increased. The overall connectivity category (i.e., high, restricted, none) concluded in each study did not differ significantly between studies that used deterministic or probabilistic models, when considering all studies (*χ* ^2^ = 0.53, df = 2, *P *=* *0.77) or when considering each of the three scales individually (*χ* ^2^ = 0.25–0.53, df = 2, *P *=* *0.77–0.88). Therefore, studies were not separated on the basis of analytical methodology when analyzed further.

The prevalence of conclusions of high, restricted and no connectivity did not differ significantly among the three most‐studied arid regions (the Australian arid zone, North American deserts, and Mediterranean Basin drylands; *χ* ^2^ = 4.18–7.19, df = 4, *P *=* *0.13–0.38). No significant differences in connectivity were found between species in the two most‐studied habitat types (rivers and springs) at the two larger spatial scales (*χ* ^2^ = 2.54–4.81, df = 4, *P *=* *0.09–0.28). In contrast, at the smallest scale, there were significantly more conclusions of low connectivity for species inhabiting spring systems than rivers (*χ* ^2^ = 9.71, df = 4, *P *<* *0.01).

**Figure 5:**
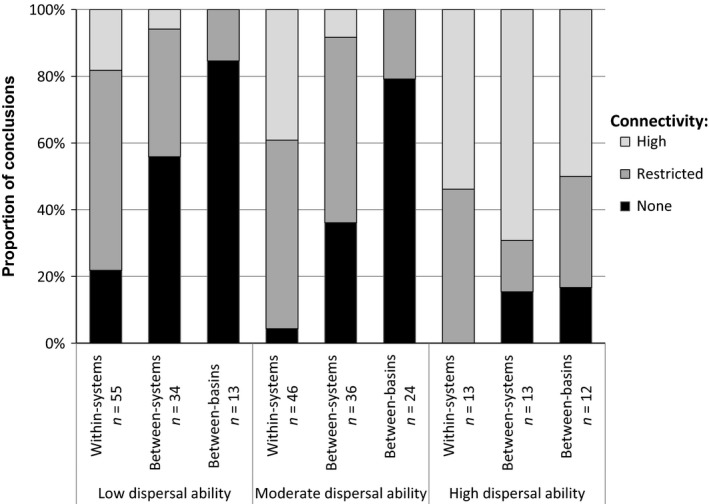
Proportion of studies of desert freshwater taxa that concluded three categories of connectivity, at three different scales, compared between species with three levels of dispersal ability.

The three most‐studied taxonomic groups (fish, crustaceans, mollusks; see Appendix S2 in Supporting Information) showed no significant differences in connectivity patterns from each other at any scale (*χ* ^2^ = 0.76–8.18, df = 4, *P *=* *0.09–0.94). However, species with higher dispersal abilities showed higher connectivity than moderately dispersing species at all three scales, while the moderate dispersers showed higher connectivity than the low dispersal ability species at all three scales (Fig. 5). These differences were significant at all three scales (*χ* ^2^ = 14.52–33.12, df = 4, *P *<* *0.01).

## Discussion

Our review of 133 studies of connectivity of aquatic fauna in desert freshwaters has shown that patterns of connectivity do not differ strongly between regions or taxa, or analytical methods used, but are strongly correlated with species’ dispersal ability and habitat (spring or river). Connectivity declined at larger scales. Few studies tested existing connectivity models, and instead, most described the levels of gene flow or population structure. Here, we discuss these results, highlight future research opportunities, and argue for analyses of connectivity in desert freshwaters that test predefined models of connectivity with probabilistic methods.

### Advances in molecular population biology

Since 1987, when the first study reviewed was published, molecular methods have advanced rapidly and many new data collection and analytical techniques have become accessible to ecologists. The general replacement of allozymes by microsatellites and sequence data typically provides greater power in connectivity studies; however, their higher costs may mean that fewer loci can be included in a project. Some papers included very few loci indeed – almost 20% were based on just a single (usually mtDNA) locus. This is problematic because single‐gene phylogenies reflect only the history of the gene (and in the case of mtDNA, the history of a maternal lineage), which may or may not mirror the population history (Edwards and Beerli 2000). In addition, a single gene might potentially be under selection or linked to genes under selection. Using multiple loci reduces these problems and can be considered independent tests of hypotheses, yielding higher sensitivity (Sunnucks 2000).

Technical advances have also allowed more marker classes to be used in studies, which has multiple benefits. The different processes governing the evolution of these markers means they can reflect different aspects of population biology and history, and different timescales. For example, a high level of subdivision was detected for the Australian spring snail *Fonscochlea accepta* in a study that used nine fast‐evolving microsatellites (Worthington Wilmer et al. 2008), whereas no subdivision was detected when using a slower evolving mtDNA marker (Murphy et al. 2010). Including multiple marker classes, notably uniparental as well as biparental, and sequence‐based as well as frequency‐based data, will also allow a stronger understanding of historical and contemporary connectivity patterns.

While recent connectivity studies have utilized a range of modern analytical methods, most connectivity research has been based on deterministic models, especially *F*_ST_. *F*_ST_ is a measure of the degree of difference between genetic samples (Wright 1951). The original model of *F*_ST_ was based on a network of panmictic subpopulations of the same constant size, connected by constant migration in migration‐drift equilibrium, with no mutation and no selection Under these conditions, gene flow is proportional to the inverse of *F*_ST_ and is unaffected by the geographic distance between populations (Wright 1951; Neigel 2002). The major assumptions of *F*_ST_ are often violated in natural systems (Marko and Hart 2011) and would seem to be especially unlikely to hold in the extreme and dynamic environments of desert freshwaters. In this environment, frequent changes in population sizes and connectivity, and accumulation of mutations in isolated populations, are expected to reduce the likelihood of the establishment of genetic equilibria.

While we found a strong shift over time toward methods that estimate gene flow with probabilistic models, some recent studies continue to use deterministic models. Unexpectedly, however, we found no significant difference between the conclusions reached via the two methodologies. This surprising result has been found elsewhere, with *F*_ST_ shown to be robust to violation of assumptions and having a strong empirical track record (Neigel 2002; Whitlock 2011). Nevertheless, there is a strong chance that deterministic models will be incorrect in some studies, and where incorrect inferences are made, these will assume that gene flow plays a greater role in preventing divergence than is the case (Marko and Hart 2011). This can have negative effects on conservation management. For example, if resources are allocated to preserving wrongly inferred gene flow, then conservation resources are wasted on neutral or even negative outcomes (e.g., outbreeding depression).

Fortunately, advanced genetic analysis methodologies, including coalescence, assignment methods, and approximate Bayesian computation, mean that it is now unnecessary to make such assumptions and allow more realistic modeling of complex connectivity scenarios (Marko and Hart 2011). These probabilistic models also offer other benefits, including for conservation. For example, estimates of divergence times can be used to determine whether the cause of divergence between populations is natural or anthropogenic, a key factor when deciding how to manage connectivity (Crandall et al. 2000). They also offer better estimates of levels of gene flow and a quantitative approach to studying connectivity, which allows optimization of conservation management.

Modern approaches also offer a range of methods to test different connectivity models. We found that these models were under‐utilized: very few papers considered a range of connectivity models, and many did not explicitly mention any model. While some studies did not focus on connectivity, many that did ignored existing landscape connectivity models, even when they had the data to test them. Of those that did test models, most tested only Isolation by Distance and/or panmixia as null hypotheses, which in many cases are unlikely to reflect the true dispersal scenarios for aquatic organisms in deserts. Because models exist to aid understanding of systems, and connectivity modeling is vital for management and conservation of threatened species and communities (Vrijenhoek 1998; McRae et al. 2008; Ferrarini 2013), the extensive under‐utilization of connectivity models represents substantial missed opportunities.

### Biases in study locations and taxa

We found strong biases in the geographic locations and taxa included in reviewed studies. The bias we detected toward studying in developed countries and “close to home” has been noted in many fields and is a major issue in field‐based biology research (Pyšek et al. 2008). Here, it restricts our understanding of connectivity in desert freshwaters. Studies in the deserts of Africa, Asia, and South America should be prioritized, and collaboration with local researchers required, as suggested in regard to phylogeographic studies by Beheregaray (2008).

The results of our literature search indicate that among those groups that were well studied, there was a bias toward larger taxa, along with a focus on endangered fish taxa (especially among the North American and Iberian studies). Given the globally threatened nature of freshwaters, and limited research resources, studies should attempt to consider multiple species, ideally representing a broad sampling of relevant life‐history traits, in order to best inform management.

### Drivers of connectivity patterns in desert freshwaters

While several connectivity models applied to freshwater systems explicitly consider geomorphology, principally drainage patterns and topography, hydrology is rarely incorporated. This may be because flows are generally constant and therefore have little effect on connectivity in the relatively well‐studied mesic regions. In contrast, hydrology is extremely variable in most arid regions (Van Etten 2009) and likely has a greater effect than geomorphology in determining the connectivity patterns of many desert freshwater species (Sheldon et al. 2010).

Hydrology can affect connectivity in a number of ways. Higher frequency, larger volume, and greater duration flows are all likely to increase connectivity. Climate change and anthropogenic disturbance scenarios largely predict flow will decrease and become more variable, leading to greater fragmentation and isolation (Woodward et al. 2010; Jaeger et al. 2014). However, few studies have been able to model these scenarios with respect to connectivity in desert freshwaters, and none of the studies included here did. Isolation by Resistance models offer an opportunity to explore the effects of hydrology: under a circuit‐theory approach, lateral hydrological connections during flood events predicted the connectivity patterns of aquatic invertebrate communities in arid Western Australia (Morán‐Ordóñez et al. 2015). Hydrology is a vital component of connectivity in desert freshwaters and requires greater consideration in future studies.

Species’ ecology is another major driver of desert freshwater connectivity patterns. While we found no significant differences in connectivity patterns among the three most‐studied taxonomic groups (fish, crustaceans, and mollusks), connectivity was significantly different between species with different dispersal abilities. Taxa with high dispersal ability showed high connectivity at all three scales, as expected, while some species with low dispersal ability showed no evidence of connectivity even at the smallest scale. While the overall pattern of decreasing connectivity at larger scales (Fig. 4) may seem an obvious result, this is not necessarily the case. Several studies found no change in connectivity across scales (e.g., Bostock et al. 2006; Stutz et al. 2010), because connectivity is dependent on the spatial scale relative to a species’ dispersal ability. Accordingly, connectivity studies of ecological communities are increasingly analyzed according to groups of taxa organized by dispersal mode or ability (Morán‐Ordóñez et al. 2015; Phillipsen et al. 2015).

Many species showed less connectivity than expected given their dispersal ability, and while this is expected to be largely due to a lack of hydrological connectivity, there may be other reasons. One is selection pressure against dispersal (Maes et al. 2013), given the often low chance of finding a better habitat in arid environments. While most studies considered their taxon's dispersal ability, some ignored dispersal altogether and considered only geomorphology and/or hydrology as drivers of connectivity. Researchers need to incorporate dispersal and other life‐history traits when comparing population connectivity.

Researchers should also consider that different connectivity models may be appropriate at different scales, locations, or habitat types. Dispersal traits are not necessarily identical for all members of a species: they may vary among locally adapted populations, and according to local environmental differences (Baguette and Van Dyck 2007; Maes et al. 2013). Several studies found differences in connectivity patterns in different parts of a species’ range, although this was always attributed to differences in structural connectivity (e.g., Carini and Hughes 2004; Murphy and Austin 2004; Huey et al. 2006). However, species may also be locally adapted to different habitats. At the within‐system scale, we observed significantly lower levels of connectivity in spring habitats than in rivers, and even no connectivity between springs separated by only a few hundred meters (e.g., Murphy et al. 2010). However, rather than resulting simply from limited hydrological connections, this pattern may reflect locally endemic species that are strongly dispersal‐limited. Such a trait may be particularly useful in arid environments, where dispersal from permanent habitats, such as springs is highly likely to result in mortality (Maes et al. 2013). In contrast, behavioral experiments have shown the opposite for one desert dweller. Desert gobies, an arid Australian fish, from isolated spring‐dwelling populations were often more dispersive than those from more‐connected river systems (K.D. Mossop, N.P. Moran, D.G. Chapple, B.B.M. Wong, unpubl. ms.), which may be a mechanism for maintaining connectivity. If dispersal success does differ between populations (e.g., between those in temporary and permanent habitats), then connectivity patterns may differ (Berendonk and Bonsall 2002), and while this was not found in any of the studies reviewed, it should be considered.

The spatial and temporal transience of many populations of desert freshwater species adds additional complexity to connectivity studies. Many taxa exist in metapopulations, with subpopulations establishing during wetter times and becoming extinct when their habitat dries (e.g., Huey et al. 2011). Such regular local extinctions and recolonization events may erase the genetic signatures of the drainage pattern, masking true connectivity patterns. When colonization events have occurred recently, equilibrium between genetic divergence and dispersal may not have been reached, meaning the assumptions of traditional genetic analyses are likely to be violated (Woods et al. 2010). Researchers should identify systems where a metapopulation structure is likely and ensure that these processes are considered when studying desert freshwater connectivity.

### Conservation implications of desert freshwater connectivity studies

Understanding connectivity is vital for the conservation of desert freshwaters, especially given the major threats that climate change and anthropogenic impacts pose in arid regions (Dudgeon et al. 2006). For example, information on hydrological connectivity is useful for identifying refugia that allow multiple taxa to persist through drought periods. These should be priority sites for conservation management and protection, as these habitats facilitate the persistence of the three recognized levels of biodiversity (genetic, species and ecosystem), especially under adverse environmental conditions (Sheldon et al. 2002, 2010; Davis et al. 2013; Costelloe and Russell 2014; Jaeger et al. 2014). However, understanding the ecology of specific taxa is also important for management. Phillipsen et al. (2015) analyzed three insect species with different dispersal abilities in North American desert streams and noted that climate change would affect each species differently. As such, each requires a different conservation management approach, and this is likely to be the case for many coexisting desert freshwater species.

While reporting connectivity patterns is clearly useful, incorporating connectivity models into studies is essential for conserving biodiversity and managing aquatic ecosystems (Hughes et al. 2013). Indeed, some models were built specifically to guide management. The Stream Hierarchy Model was developed for threatened North American desert fishes and advocates management that maintains natural connectivity patterns and levels in order to maintain populations and their genetic diversity (Meffe and Vrijenhoek 1988). Because many desert freshwater species exist as metapopulations in transient habitats, there are often no habitats that require constant protection. Instead, protection of the processes driving connectivity is required, especially hydrological connectivity. In contrast, species that conform to the Death Valley Model exist as genetically distinct, isolated populations and should be maintained as such (Meffe and Vrijenhoek 1988). This model is especially applicable to short‐range endemic species, such as the spring amphipods of central Australia, and their conservation requires both protection of their typically small habitats and prevention of any artificial gene flow (Murphy et al. 2013). On the other hand, panmictic species, such as those able to disperse widely via temporary connections during floods, may require protection of only a small number of key habitats (Moritz 1999). Connectivity models can inform a range of management decisions, including how limited conservation resources are allocated, which aquatic habitats are prioritized for greatest protection, and the optimal management actions under a given set of goals and constraints.

## Conclusions

How populations persist is a question that has fascinated scientists for decades (Mari et al. 2014). Answering this question is critical for the threatened aquatic ecosystems of the world's deserts. Persistence in these ecosystems often relies on connectivity, and we have shown that, regardless of location or taxonomic classification, this is driven by a species’ ecology and the hydrology of its habitat. Untangling the effects of these drivers is complex, and we have conducted the first “health‐check” of research in this field. We advocate obtaining DNA sequence data from mitochondrial and multiple nuclear markers, and applying an integrated range of approaches, including coalescent analyses and approximate Bayesian computations, to estimate gene flow and other key population parameters at different temporal and spatial scales. These estimates can be used to test a range of explicit models of connectivity. Such an approach will give greater power and accuracy and minimize the number of assumptions. We draw attention to the advantages of greater utilization of existing connectivity models, and extending these to more realistically address questions in specific regions (Morán‐Ordóñez et al. 2015). Finally, studies are urgently needed on the desert freshwaters of Africa, Asia, and South America. These systems have received little research attention, yet face the same threats as other desert freshwaters, which are among the most threatened ecosystems on the planet.

## Conflict of Interest

None declared.

## Supporting information


**Appendix S1.** Dataset of included studies of desert freshwater connectivity.
**Appendix S2.** Graphical comparison of connectivity levels among taxonomic groupings.
