# Supplementary material for: Swimming through sand: connectivity of aquatic fauna in deserts
Source: Ecol Evol. 2015 Oct 23;5(22):5252–64. doi: 10.1002/ece3.1741 (PMC6102528; doi:10.1002/ece3.1741)
Supplement: Supplementary file 1 — Appendix S1. Dataset of included studies of desert freshwater connectivity. Appendix S2. Graphical comparison of connectivity levels among taxonomic groupings. [file ECE3-5-5252-s001.docx]

**SWIMMING THROUGH SAND: CONNECTIVITY OF AQUATIC FAUNA IN DESERTS**

Ashley L Murphy, Alexandra Pavlova, Ross Thompson, Jenny Davis and Paul Sunnucks

*Ecology & Evolution*

**Appendix S1**. Dataset of 133 desert freshwater connectivity studies reviewed to give an overview of the trends in the field, with selected details of study systems, methodologies and conclusions noted. Details include: Species – species names as provided in source; Class – taxonomic class of organism; Disp – predicted dispersal ability of organism (low, moderate, high – see Methods for definitions); Study Location – country where species sampled (number only where more than two countries sampled); Habitat Type – habitat type sampled (pools, rivers, springs or multiple); Markers Used – class/es of molecular marker used in study (allozymes (Allo), amplified fragment length polymorphisms (AFLP), nuclear DNA sequences (nDNA), microsatellites (msats), mitochondrial DNA (mtDNA), randomly amplified polymorphic DNA (RAPD), restricted fragment length polymorphisms (RFLP) and single-primer amplification reaction (SPAR)); Analytical Method – method used for analyses of genetic data (deterministic or probabilistic*); Connectivity Model Concluded – conclusion of connectivity model or description of gene flow (Panmixia, Isolation By Distance (IBD), Stream Hierarchy Model (SHM), Death Valley Model (DVM), no, restricted or high gene flow (GF)), at each of three scales*. *See Methods section for definitions of these terms. A full reference list of sources is provided below.

|  |  |  |  |  |  |  |  | **Connectivity Model Concluded** | | |
| --- | --- | --- | --- | --- | --- | --- | --- | --- | --- | --- |
| **Source** | **Species** | **Class** | **Disp** | **Study Location** | **Habitat Type** | **Markers Used** | **Analytical Method** | **Within Systems** | **Between Systems** | **Between Basins** |
| Echelle *et al.* 1987 | *Cyprinodon bovinus* | Osteichthyes | Low | USA | Multiple | Allo | Deterministic | High GF | – | – |
|  | *Cyprinodon elegans* | Osteichthyes | Low | USA | Springs | Allo | Deterministic | IBD | – | – |
|  | *Cyprinodon pecosensis* | Osteichthyes | Low | USA | Rivers | Allo | Deterministic | High GF | – | – |
|  | *Cyprinodon tularosa* | Osteichthyes | Low | USA | Springs | Allo | Deterministic | Low GF | – | – |
| Echelle *et al.* 1989 | *Gambusia nobilis* | Osteichthyes | Mod | USA | Springs | Allo | Deterministic | High GF | Restricted GF | – |
| Ponder *et al.* 1995 | *Fonscochlea accepta* | Gastropoda | Mod | Australia | Springs | Allo | Deterministic | IBD | – | – |
|  | *Fonscochlea aquatica* | Gastropoda | Mod | Australia | Springs | Allo | Deterministic | IBD | – | – |
|  | *Fonscochlea variabilis* | Gastropoda | Mod | Australia | Springs | Allo | Deterministic | IBD | – | – |
|  | *Fonscochlea zeidleri* | Gastropoda | Mod | Australia | Springs | Allo | Deterministic | IBD | – | – |
|  | *Trochidrobia punicea* | Gastropoda | Low | Australia | Springs | Allo | Deterministic | IBD | – | – |
|  | *Trochidrobia smithi* | Gastropoda | Low | Australia | Springs | Allo | Deterministic | IBD | – | – |
| Quattro *et al.* 1996 | *Poeciliopsis occidentalis* | Osteichthyes | Mod | USA | Rivers | mtDNA | Deterministic | High GF | Low GF | Low GF |
| Tibbets & Dowling 1996 | *Tiaroga cobitis* | Osteichthyes | Mod | USA | Rivers | Allo, RFLP | Deterministic | No GF | No GF | – |
|  | *Meda fulgida* | Osteichthyes | Mod | USA | Rivers | Allo, RFLP | Deterministic | Restricted GF | No GF | – |
|  | *Agosia chrysogaster* | Osteichthyes | Mod | USA | Rivers | Allo, RFLP | Deterministic | Restricted GF | No GF | – |
| Davies *et al.* 1997 | *Branchinecta sandiegonensis* | Branchiopoda | Low | USA | Pools | Allo | Deterministic | – | Low GF | – |
| Thomas *et al.* 1997 | *Hyalella azteca* | Malacostraca | Low | USA | Pools | RAPD | Deterministic | Restricted GF | – | – |
|  | *Hyalella montezuma* | Malacostraca | Low | USA | Pools | RAPD | Deterministic | IBD | – | – |
|  |  |  |  |  |  |  |  | **Connectivity Model Concluded** | | |
| **Source** | **Species** | **Class** | **Disp** | **Study Location** | **Habitat Type** | **Markers Used** | **Analytical Method** | **Within Systems** | **Between Systems** | **Between Basins** |
| Viard *et al.* 1997 | *Bulinus truncatu* | Gastropoda | Low | 8 Countries | Rivers | Msats | Deterministic | IBD | Restricted GF | No GF |
| Duvernell & Turner 1998 | *Cyprinodon nevadensis* | Osteichthyes | Low | USA | Rivers | mtDNA | Deterministic | Restricted GF | No GF | – |
|  | *Cyprinodon salinus* | Osteichthyes | Low | USA | Rivers | mtDNA | Deterministic | No GF | – | – |
| Johnson & Jordan 2000 | *Gila copei* | Osteichthyes | Mod | USA | Rivers | mtDNA | Deterministic | – | – | No GF |
| Miller *et al.* 2000 | *Oxylema haydeni* | Gastropoda | Low | USA | Springs | AFLP, mtDNA | Deterministic | No GF | – | – |
| Mesquita *et al.* 2001 | *Chondrostoma lusitanicum* | Osteichthyes | Mod | Portugal | Rivers | mtDNA, RFLP | Deterministic | High GF | – | Low GF |
| Cook *et al.* 2002 | *Macrobrachium australiense* | Malacostraca | Mod | Australia | Rivers | Allo, mtDNA | Deterministic | Panmixia | DVM | DVM |
| Johnson, 2002 | *Gila atraria* | Osteichthyes | Mod | USA | Rivers | mtDNA | Deterministic | – | IBD | No GF |
| Miller *et al.* 2002 | *Ambrysus thermarum* | Insecta | Low | USA | Rivers | AFLP | Deterministic | High GF | High GF | – |
|  | *Psephenus montanus* | Insecta | Low | USA | Rivers | AFLP | Deterministic | No GF | No GF | – |
| Nielsen & Sage 2002 | *Oncorhynchus clarki* | Osteichthyes | Mod | USA | Rivers | Msats | Deterministic | – | No GF | – |
| Douglas *et al.* 2003 | *Catostomus latipinnis* | Osteichthyes | Mod | USA | Rivers | mtDNA | Probabilistic | High GF | High GF | – |
| Hughes & Hillyer 2003 | *Cherax destructor* | Malacostraca | Mod | Australia | Rivers | mtDNA | Deterministic | High GF | Low GF | Low GF |
| Whitehead *et al.* 2003 | *Catostomus occidentalis* | Osteichthyes | Mod | USA | Rivers | AFLP, Msats | Deterministic | IBD | Restricted GF | – |
| Carini & Hughes 2004 | *Macrobrachium australiense* | Malacostraca | Mod | Australia | Rivers | mtDNA | Probabilistic | Restricted GF | Restricted GF | No GF |
| Gervasio *et al.* 2004 | *Gammarus pecos* | Malacostraca | Low | USA | Springs | Allo | Deterministic | IBD | – | – |
| Gow *et al.* 2004 | *Bulinus forskalii* | Gastropoda | Low | Cameroon | Multiple | Msats | Probabilistic | – | High GF | – |
| Hughes *et al.* 2004 | *Velesunio* spp. A | Bivalvia | Mod | Australia | Rivers | Allo, mtDNA | Probabilistic | Restricted GF | No GF | – |
|  | *Velesunio* spp. B | Bivalvia | Mod | Australia | Rivers | Allo, mtDNA | Probabilistic | Restricted GF | – | – |
|  | *Velesunio* spp. C | Bivalvia | Mod | Australia | Rivers | Allo, mtDNA | Probabilistic | Restricted GF | Low GF | No GF |
|  | *Velesunio* spp. D | Bivalvia | Mod | Australia | Rivers | Allo, mtDNA | Probabilistic | Restricted GF | – | – |
| Martin & Wilcox 2004 | *Cyprinodon nevadensis* | Osteichthyes | Low | USA | Springs | Msats | Deterministic | Restricted GF | Low GF | – |
| Mock *et al.* 2004 | *Anodonta californiensis* | Bivalvia | Mod | USA | Multiple | AFLP, mtDNA | Deterministic | – | No GF | – |
| Moline *et al.* 2004 | *Nymphophilus minckleyi* | Gastropoda | Low | Mexico | Springs | Allo | Deterministic | High GF | – | – |
| Murphy & Austin 2004 | *Macrobrachium australiense* | Malacostraca | Mod | Australia | Rivers | mtDNA | Deterministic | – | Restricted GF | No GF |
| Nguyen *et al.* 2004 | *Cherax destructor* | Malacostraca | Mod | Australia | Rivers | mtDNA | Deterministic | Restricted GF | Low GF | No GF |
| Alo & Turner 2005 | *Hybognathus amarus* | Osteichthyes | Mod | USA | Rivers | Msats, mtDNA | Deterministic | High GF | – | – |
| Hershler *et al.* 2005 | *Tryonia porrecta* | Gastropoda | Low | USA | Springs | Allo, mtDNA | Deterministic | – | – | Restricted GF |
| Johnson 2005 | *Mexipyrgus churinceanus* | Gastropoda | Low | Mexico | Multiple | mtDNA | Probabilistic | IBD | No GF | – |
| Mamuris *et al.* 2005 | *Ladigesocypris ghigii* | Osteichthyes | Mod | Greece | Rivers | RAPD, RFLP | Deterministic | High GF | – | No GF |
| Mesquita *et al.* 2005 | *Squalius aradensis* | Osteichthyes | Mod | Portugal | Rivers | Msats, mtDNA | Deterministic | – | Restricted GF | No GF |
| Mock & Miller 2005 | *Iotichthys phlegethontis* | Osteichthyes | Low | USA | Springs | AFLP, mtDNA | Deterministic | Restricted GF | No GF | – |
| Bostock *et al.* 2006 | *Leiopotherapon unicolor* | Osteichthyes | High | Australia | Rivers | Allo, mtDNA | Deterministic | High GF | High GF | High GF |
| Carini & Hughes 2006 | *Notopala sublineata* | Gastropoda | Low | Australia | Rivers | Allo, mtDNA | Probabilistic | Restricted GF | Restricted GF | No GF |
| Carini et al 2006 | *Macrobrachium australiense* | Malacostraca | Mod | Australia | Rivers | mtDNA | Deterministic | High GF | – | – |
|  | *Notopala sublineata* | Gastropoda | Low | Australia | Rivers | Allo, mtDNA | Deterministic | High GF | – | – |
|  |  |  |  |  |  |  |  | **Connectivity Model Concluded** | | |
| **Source** | **Species** | **Class** | **Disp** | **Study Location** | **Habitat Type** | **Markers Used** | **Analytical Method** | **Within Systems** | **Between Systems** | **Between Basins** |
| Carson & Dowling 2006 | *Cyprinodon atrorus* | Osteichthyes | Low | Mexico | Multiple | nDNA, mtDNA | Deterministic | Restricted GF | – | No GF |
|  | *Cyprinodon bifasciatus* | Osteichthyes | Low | Mexico | Multiple | nDNA, mtDNA | Deterministic | Restricted GF | – | No GF |
| Cegelski *et al.* 2006 | *Oncorhynchus clarkii* | Osteichthyes | Mod | USA | Rivers | Msats | Probabilistic | Multiple | Restricted GF | – |
| Colgan *et al.* 2006 | *Caldicochlea globosa* | Gastropoda | Low | Australia | Springs | mtDNA | Deterministic | No GF | – | – |
|  | *Caldicochlea harrisi* | Gastropoda | Low | Australia | Springs | mtDNA | Deterministic | No GF | – | – |
| Huey *et al.* 2006 | *Neosilurus hyrtlii* | Osteichthyes | Mod | Australia | Rivers | Allo, mtDNA, Msats | Probabilistic | Panmixia | No GF | No GF |
|  | *Porochilus argenteus* | Osteichthyes | Mod | Australia | Rivers | mtDNA, Msats | Probabilistic | Panmixia | – | – |
| Hughes & Hillyer 2006 | *Nematolosa erebi* | Osteichthyes | Mod | Australia | Rivers | Allo, mtDNA | Probabilistic | Restricted GF | No GF | No GF |
|  | *Retropinna semoni* | Osteichthyes | Low | Australia | Rivers | Allo, mtDNA | Probabilistic | SHM | No GF | No GF |
| Miller *et al.* 2006 | *Valvata utahensis* | Gastropoda | Low | USA | Rivers | AFLP, mtDNA | Deterministic | High GF | IBD | – |
| Mock *et al.* 2006 | *Catostomus ardens* | Osteichthyes | Mod | USA | Rivers | mtDNA, AFLP | Deterministic | – | Restricted GF | No GF |
| Bernardi *et al.* 2007 | *Fundulus lima* | Osteichthyes | Low | Mexico | Springs | mtDNA | Probabilistic | – | No GF | – |
| Hulsmans *et al.* 2007 | *Branchipodopsis wolfi* | Branchiopoda | Mod | Botswana | Pools | Allo | Deterministic | IBD | IBD | – |
| Zickovich & Bohonak 2007 | *Hyalella azteca* | Malacostraca | Low | USA | Rivers | mtDNA | Probabilistic | IBD | – | – |
| Huey *et al*. 2008 | *Neosilurus hyrtlii* | Osteichthyes | Mod | Australia | Rivers | Allo, mtDNA, msats | Probabilistic | SHM | SHM | No GF |
| Masci *et al*. 2008 | *Nematalosa erebi* | Osteichthyes | Mod | Australia | Rivers | mtDNA | Probabilistic | IBD | IBD | No GF |
|  | *Macrobrachium australiense* | Malacostraca | Mod | Australia | Rivers | mtDNA | Probabilistic | – | – | No GF |
| Munoz *et al.* 2008 | *Artemia salina* | Anacostraca | High | 8 Countries | Pools | mtDNA | Deterministic | – | – | No GF |
| Pamponet *et al.* 2008 | *Astyanax* aff. *bimaculatus* | Osteichthyes | Mod | Brazil | Rivers | RAPD, SPAR | Deterministic | – | No GF | No GF |
| Sousa *et al.* 2008 | *Chondrostoma lusitanicum* | Osteichthyes | Mod | Portugal | Rivers | mtDNA, Msats | Deterministic | – | Restricted GF | No GF |
| Worthington Wilmer *et al.* 2008 | *Fonscochlea accepta* | Gastropoda | Mod | Australia | Springs | Msats | Probabilistic | High GF | IBD | – |
| Loftis *et al.* 2009 | *Cyprinodon eremus* | Osteichthyes | Low | Mexico, USA | Rivers | Msats | Deterministic | IBD | – | – |
| Murphy *et al.* 2009 | *Austrochiltonia dalhousiensis* | Malacostraca | Low | Australia | Springs | Allo, mtDNA | Deterministic | Restricted GF | – | – |
|  | *Austrochiltonia* spp. A | Malacostraca | Low | Australia | Springs | Allo, mtDNA | Deterministic | Restricted GF | No GF | – |
|  | *Austrochiltonia* spp. B | Malacostraca | Low | Australia | Springs | Allo, mtDNA | Deterministic | Restricted GF | No GF | – |
|  | *Austrochiltonia* spp. C | Malacostraca | Low | Australia | Springs | Allo, mtDNA | Deterministic | Restricted GF | Restricted GF | – |
|  | *Austrochiltonia* spp. F | Malacostraca | Low | Australia | Springs | Allo, mtDNA | Deterministic | Restricted GF | – | – |
|  | *Phreatochiltonia anophthalma* | Malacostraca | Low | Australia | Springs | Allo, mtDNA | Deterministic | No GF | – | – |
| Phillipsen & Metcalf 2009 | *Pseudacris cadaverina* | Amphibia | Low | USA | Rivers | mtDNA | Deterministic | Restricted GF | Restricted GF | No GF |
| Pritchard *et al.* 2009 | *Oncorhynchus clarkii* | Osteichthyes | Mod | USA | Rivers | mtDNA, Msats | Probabilistic | SHM | No GF | No GF |
| Sei *et al.* 2009 | *Gambusia nobilis* | Osteichthyes | Mod | USA | Springs | Allo | Deterministic | Restricted GF | – | – |
|  | *Gammarus pecos* | Malacostraca | Low | USA | Springs | Allo | Deterministic | IBD | – | – |
| Seidel *et al.* 2009 | *Gammarus* sp. "roswell" | Malacostraca | Low | USA | Springs | mtDNA | Deterministic | Restricted GF | – | – |
|  | *Gammarus* sp. "toyah" | Malacostraca | Low | USA | Springs | mtDNA | Deterministic | Restricted GF | – | – |
|  |  |  |  |  |  |  |  | **Connectivity Model Concluded** | | |
| **Source** | **Species** | **Class** | **Disp** | **Study Location** | **Habitat Type** | **Markers Used** | **Analytical Method** | **Within Systems** | **Between Systems** | **Between Basins** |
| Wang 2009 | *Bufo exsul* | Amphibia | Low | USA | Springs | Msats | Probabilistic | Restricted GF | – | – |
| Faulks *et al.* 2010 | *Macquaria ambigua* | Osteichthyes | Mod | Australia | Rivers | Msats | Probabilistic | High GF | Restricted GF | No GF |
| Billman *et al.* 2010 | *Rhinichthys osculus* | Osteichthyes | Mod | USA | Rivers | mtDNA | Deterministic | – | – | Low GF |
| Henriques *et al.* 2010 | *Squalius torgalensis* | Osteichthyes | Mod | Portugal | Rivers | mtDNA, Msats | Probabilistic | High GF | – | – |
| Jungels *et al.* 2010 | *Bufo cognata* | Amphibia | Mod | USA | Rivers | Msats | Deterministic | IBD | IBD | – |
| Korn *et al.* 2010 | *Triops baeticus* | Branchiopoda | High | Portugal, Spain | Multiple | mtDNA | Deterministic | – | High GF | High GF |
|  | *Triops gadensis* | Branchiopoda | High | Spain | Multiple | mtDNA | Deterministic | – | High GF | High GF |
|  | *Triops vicenticus* | Branchiopoda | High | Portugal | Multiple | mtDNA | Deterministic | – | – | No GF |
| Martin 2010 | *Cyprinodon nevadensis* | Osteichthyes | Low | USA | Springs | mtDNA, Msats | Probabilistic | High GF | Low GF | – |
| Mock *et al.* 2010 | *Anodonta californiensis/ nuttalliana* | Bivalvia | Mod | USA | Rivers | Msats, mtDNA | Deterministic | – | No GF | No GF |
| Murphy *et al.* 2010 | *Fonscochlea accepta* | Gastropoda | Mod | Australia | Springs | mtDNA | Probabilistic | High GF | High GF | – |
|  | *Ngarwa dirge* | Ostracoda | High | Australia | Springs | mtDNA | Probabilistic | High GF | High GF | – |
|  | *Phreatomerus latipes* | Malacostraca | Low | Australia | Springs | mtDNA | Probabilistic | High GF | SHM | – |
|  | *Wangiannachiltonia guzikae* | Malacostraca | Low | Australia | Springs | mtDNA | Probabilistic | No GF | No GF | – |
| Sousa *et al.* 2010 | *Iberochondrostoma almacai* | Osteichthyes | Low | Portugal | Rivers | Msats, mtDNA | Deterministic | Panmixia | Restricted GF | – |
| Stutz *et al.* 2010 | *Hyalella azteca* | Malacostraca | Low | USA | Springs | mtDNA, nDNA | Deterministic | No GF | No GF | No GF |
| Woods *et al.* 2010 | *Retropinna semoni* | Osteichthyes | Low | Australia | Rivers | Allo, Msats, mtDNA | Probabilistic | Restricted GF | Low GF | – |
| Chaves-Campos *et al.* 2011 | *Palaemonetes suttkusi* | Malacostraca | Mod | Mexico | Springs | mtDNA | Probabilistic | Restricted GF | – | Low GF |
| Huey *et al.* 2011 | *Macquaria ambigua* | Osteichthyes | Mod | Australia | Rivers | Msats | Deterministic | Restricted GF | – | – |
|  | *Tandanus tandanus* | Osteichthyes | Mod | Australia | Rivers | Msats | Deterministic | Panmixia | – | – |
|  | *Macrobrachium australiense* | Malacostraca | Mod | Australia | Rivers | Msats | Deterministic | Restricted GF | – | – |
| Morales *et al.* 2011 | *Orestias ascotanensis* | Osteichthyes | Mod | Chile | Springs | mtDNA | Probabilistic | Restricted GF | No GF | – |
| Small *et al.* 2011 | *Oncorhynchus tshawytscha* | Osteichthyes | Mod | USA | Rivers | Msats | Probabilistic | High GF | Low GF | – |
| Guzik *et al.* 2012 | *Phreatomerus latipes* | Malacostraca | Low | Australia | Springs | Allo, mtDNA | Probabilistic | Restricted GF | No GF | – |
| Lopes-Cunha *et al.* 2012 | *Iberochondrostoma lemmingii* | Osteichthyes | Low | Portugal, Spain | Rivers | Msats, mtDNA | Deterministic | – | Restricted GF | No GF |
| McGaugh 2012 | *Apalone atra* | Reptilia | Low | Mexico | Multiple | Msats | Deterministic | – | No GF | – |
| Murphy *et al.* 2012 | *Trochidrobia minuta* | Gastropoda | Low | Australia | Springs | mtDNA, nDNA | Probabilistic | No GF | No GF | – |
|  | *Trochidrobia punicea* | Gastropoda | Low | Australia | Springs | mtDNA, nDNA | Probabilistic | No GF | No GF | – |
|  | *Trochidrobia smithi* | Gastropoda | Low | Australia | Springs | mtDNA, nDNA | Probabilistic | No GF | No GF | – |
| Schwenter *et al.* 2012 | *Limnadopsis birchii* | Branchiopoda | High | Australia | Rivers | mtDNA, nDNA | Probabilistic | IBD | Panmixia | IBD |
|  | *Limnadopsis paratatei* | Branchiopoda | High | Australia | Rivers | mtDNA, nDNA | Probabilistic | Panmixia | Panmixia | Panmixia |
|  | *Limnadopsis parvispinus* | Branchiopoda | High | Australia | Rivers | mtDNA, nDNA | Probabilistic | Multiple | Panmixia | IBD |
|  | *Limnadopsis tatei* | Branchiopoda | High | Australia | Rivers | mtDNA, nDNA | Probabilistic | Panmixia | Panmixia | Panmixia |
| Bartakova *et al.* 2013 | *Nothobranchius furzeri* | Osteichthyes | Low | Mozambique | Pools | mtDNA, Msats | Probabilistic | IBD | – | IBD |
| Coghill *et al.* 2013 | *Lepomis megalotis* | Osteichthyes | Mod | Mexico | Rivers | mtDNA | Probabilistic | – | Low GF | No GF |
|  |  |  |  |  |  |  |  | **Connectivity Model Concluded** | | |
| **Source** | **Species** | **Class** | **Disp** | **Study Location** | **Habitat Type** | **Markers Used** | **Analytical Method** | **Within Systems** | **Between Systems** | **Between Basins** |
| Hopken *et al.* 2013 | *Catostomus discobolus* | Osteichthyes | Mod | USA | Rivers | mtDNA, Msats | Probabilistic | SHM | SHM | No GF |
| Nguema *et al.* 2013 | *Biomphalaria pfeifferi* | Gastropoda | Low | Oman | Rivers | Msats | Probabilistic | Restricted GF | No GF | No GF |
| Murphy *et al.* 2013 | *Wangiannachiltonia guzikae* | Malacostraca | Low | Australia | Springs | mtDNA, nDNA | Probabilistic | DVM | DVM | – |
| Van Leeuwen *et al.* 2013 | *Physa acuta* | Gastropoda | High | Spain | Springs | Msats | Probabilistic | – | IBD | – |
| Robertson *et al.* 2014 | *Wangiannachiltonia guzikae* | Malacostraca | Low | Australia | Springs | Msats | Probabilistic | SHM | – | – |
| Schwentner *et al.* 2014 | *Eocyzicus* species | Branchiopoda | High | Australia | Rivers | mtDNA, nDNA | Deterministic | – | High GF | High GF |
| Sousa-Santos *et al.* 2014a | *Anaecypris hispanica* | Osteichthyes | Low | Portugal | Rivers | mtDNA, nDNA | Probabilistic | Restricted GF | No GF | No GF |
| Sousa-Santos *et al.* 2014b | *Iberochondrostoma olisiponensis* | Osteichthyes | Low | Portugal | Rivers | nDNA, mtDNA | Deterministic | No GF | – | – |
| Velo-Anton *et al.* 2014 | *Crocodylus suchus* | Reptilia | Mod | Mauritania | Rivers | mtDNA, Msats | Probabilistic | High GF | Restricted GF | – |
| Phillipsen *et al.* 2015 | *Abedus herberti* | Insecta | Low | USA | Rivers | Msats | Deterministic | – | No GF | – |

**References**

Alo, D. & Turner, T.F. (2005) Effects of habitat fragmentation on effective population size in the endangered Rio Grande silvery minnow. *Conservation Biology*, **19**, 1138-1148.

Bartakova, V., Reichard, M., Janko, K., Polacik, M., Blazek, R., Reichwald, K., Cellerino, A. & Bryja, J. (2013) Strong population genetic structuring in an annual fish, Nothobranchius furzeri, suggests multiple savannah refugia in southern Mozambique. *BMC Evolutionary Biology*, **13**

Bernardi, G., Ruiz-Campos, G. & Camarena-Rosales, F. (2007) Genetic isolation and evolutionary history of oases populations of the Baja California killifish, *Fundulus lima*. *Conservation Genetics*, **8**, 547-554.

Billman, E.J., Lee, J.B., Young, D.O., McKell, M.D., Evans, R.P. & Shiozawa, D.K. (2010) Phylogenetic divergence in a desert fish: differentiation of speckled dace within the Bonneville, Lahontan, and upper Snake River basins. *Western North American Naturalist*, **70**, 39-47.

Bostock, B.M., Adams, M., Laurenson, L.J.B. & Austin, C.M. (2006) The molecular systematics of *Leiopotherapon unicolor* (Gunther, 1859): testing for cryptic speciation in Australia's most widespread freshwater fish. *Biological Journal of the Linnean Society*, **87**, 537-552.

Carini, G. & Hughes, J. (2004) Population structure of *Macrobrachium australiense* (Decapoda: Palaemonidae) in Western Queensland, Australia: the role of contemporary and historical processes. *Heredity*, **93**, 350-363.

Carini, G. & Hughes, J.M. (2006) Subdivided population structure and phylogeography of an endangered freshwater snail, *Notopala sublineata* (Conrad, 1850) (Gastropoda: Viviparidae), in Western Queensland, Australia. *Biological Journal of the Linnean Society*, **88**, 1-16.

Carini, G., Hughes, J.M. & Bunn, S.E. (2006) The role of waterholes as 'refugia' in sustaining genetic diversity and variation of two freshwater species in dryland river systems (Western Queensland, Australia). *Freshwater Biology*, **51**, 1434-1446.

Carson, E.W. & Dowling, T.E. (2006) Influence of hydrogeographic history and hybridization on the distribution of genetic variation in the pupfishes *Cyprinodon atrorus* and *C. bifasciatus*. *Molecular Ecology*, **15**, 667-679.

Cegelski, C.C., Campbell, M.R., Meyer, K.A. & Powell, M.S. (2006) Multiscale genetic structure of Yellowstone cutthroat trout in the upper Snake River basin. *Transactions of the American Fisheries Society*, **135**, 711-726.

Chaves-Campos, J., Johnson, S.G., de Leon, F.J.G. & Hulsey, C.D. (2011) Phylogeography, genetic structure, and gene flow in the endemic freshwater shrimp *Palaemonetes suttkusi* from Cuatro Cinegas, Mexico. *Conservation Genetics*, **12**, 557-567.

Coghill, L.M., Hulsey, C.D., Chaves-Campos, J., de Leon, F.J.G. & Johnson, S.G. (2013) Phylogeography and conservation genetics of a distinct lineage of sunfish in the Cuatro Ciénegas Valley of Mexico. *PloS One*, **8**, e77013.

Colgan, D., Ponder, W. & Da Costa, P. (2006) Mitochondrial DNA variation in an endemic aquatic snail genus, *Caldicochlea* (Hydrobiidae; Caenogastropoda) in Dalhousie Springs, an Australian arid-zone spring complex. *Molluscan Research*, **26**, 8-18.

Cook, B.D., Bunn, S.E. & Hughes, J.M. (2002) Genetic structure and dispersal of *Macrobrachium australiense* (Decapoda: Palaemonidae) in western Queensland, Australia. *Freshwater Biology*, **47**, 2098-2112.

Davies, C.P., Simovich, M.A. & Hathaway, S.A. (1997) Population genetic structure of a California endemic branchiopod, *Branchinecta sandiegonensis*. *Hydrobiologia*, **359**, 149-158.

Douglas, M.R., Brunner, P.C. & Douglas, M.E. (2003) Drought in an evolutionary context: molecular variability in Flannelmouth Sucker (*Catostomus latipinnis*) from the Colorado River Basin of western North America. *Freshwater Biology*, **48**, 1254-1273.

Duvernell, D.D. & Turner, B.J. (1998) Evolutionary genetics of death valley pupfish populations: mitochondrial DNA sequence variation and population structure. *Molecular Ecology*, **7**, 279-288.

Echelle, A.A., Echelle, A.F. & Edds, D.R. (1987) Population-structure of 4 pupfish species (Cyprinodontidae, *Cyprinodon*) from the Chihuahuan Desert region of New Mexico and Texas - allozymic variation. *Copeia*, 668-681.

Echelle, A.F., Echelle, A.A. & Edds, D.R. (1989) Conservation genetics of a spring-dwelling desert fish, the Pecos Gambusia (*Gambusia nobilis*, Poeciliidae). *Conservation Biology*, **3**, 159-169.

Faulks, L.K., Gilligan, D.M. & Beheregaray, L.B. (2010) Islands of water in a sea of dry land: hydrological regime predicts genetic diversity and dispersal in a widespread fish from Australia's arid zone, the golden perch (*Macquaria ambigua*). *Molecular Ecology*, **19**, 4723-4737.

Gervasio, V., Berg, D.J., Lang, B.K., Allan, N.L. & Guttman, S.I. (2004) Genetic diversity in the Gammarus pecos species complex: Implications for conservation and regional biogeography in the Chihuahuan Desert. *Limnology and Oceanography*, **49**, 520-531.

Gow, J., Noble, L., Rollinson, D., Mimpfoundi, R. & Jones, C. (2004) Breeding system and demography shape population genetic structure across ecological and climatic zones in the African freshwater snail, *Bulinus forskalii* (Gastropoda, Pulmonata), intermediate host for schistosomes. *Molecular Ecology*, **13**, 3561-3573.

Guzik, M.T., Adams, M.A., Murphy, N.P., Cooper, S.J.B. & Austin, A.D. (2012) Desert springs: Deep phylogeographic structure in an ancient endemic crustacean (*Phreatomerus latipes*). *Plos One*, **7**, e37642.

Henriques, R., Sousa, V. & Coelho, M.M. (2010) Migration patterns counteract seasonal isolation of *Squalius torgalensis*, a critically endangered freshwater fish inhabiting a typical Circum-Mediterranean small drainage. *Conservation Genetics*, **11**, 1859-1870.

Hershler, R., Mulvey, M. & LIU, H.P. (2005) Genetic variation in the desert springsnail (*Tryonia porrecta*): implications for reproductive mode and dispersal. *Molecular Ecology*, **14**, 1755-1765.

Hopken, M.W., Douglas, M.R. & Douglas, M.E. (2013) Stream hierarchy defines riverscape genetics of a North American desert fish. *Molecular Ecology*, **22**, 956-971.

Huey, J.A., Hughes, J.M. & Baker, A.M. (2006) Patterns of gene flow in two species of eel-tailed catfish, *Neosilurus hyrtlii* and *Porochilus argenteus* (Siluriformes: Plotosidae), in western Queensland's dryland rivers. *Biological Journal of the Linnean Society*, **87**, 457-467.

Huey, J.A., Baker, A. & Hughes, J. (2008) The effect of landscape processes upon gene flow and genetic diversity in an Australian freshwater fish, *Neosilurus hyrtlii*. *Freshwater Biology*, **53**, 1393-1408.

Huey, J.A., Schmidt, D.J., Balcombe, S.R., Marshall, J.C. & Hughes, J.M. (2011) High gene flow and metapopulation dynamics detected for three species in a dryland river system. *Freshwater Biology*, **56**, 2378-2390.

Hughes, J., Baker, A.M., Bartlett, C., Bunn, S., Goudkamp, K. & Somerville, J. (2004) Past and present patterns of connectivity among populations of four cryptic species of freshwater mussels *Velesunio* spp. (Hyriidae) in central Australia. *Molecular Ecology*, **13**, 3197-3212.

Hughes, J.M. & Hillyer, M.J. (2003) Patterns of connectivity among populations of *Cherax destructor* (Decapoda: Parastacidae) in western Queensland, Australia. *Marine and Freshwater Research*, **54**, 587-596.

Hughes, J.M. & Hillyer, M.J. (2006) Mitochondrial DNA and allozymes reveal high dispersal abilities and historical movement across drainage boundaries in two species of freshwater fishes from inland rivers in Queensland, Australia. *Journal of Fish Biology*, **68**, 270-291.

Hulsmans, A., Moreau, K., De Meester, L., Riddoch, B.J. & Brendonck, L. (2007) Direct and indirect measures of dispersal in the fairy shrimp *Branchipodopsis wolfi* indicate a small-scale isolation-by-distance pattern. *Limnology and Oceanography*, **52**, 676-684.

Johnson, J.B. (2002) Evolution after the flood: Phylogeography of the desert fish Utah chub. *Evolution*, **56**, 948-960.

Johnson, J.B. & Jordan, S. (2000) Phylogenetic divergence in leatherside chub (*Gila copei*) inferred from mitochondrial cytochrome *b* sequences. *Molecular Ecology*, **9**, 1029-1035.

Johnson, S.G. (2005) Age, phylogeography and population structure of the microendemic banded spring snail, *Mexipyrgus churinceanus*. *Molecular Ecology*, **14**, 2299-2311.

Jungels, J.M., Griffis-Kyle, K.L. & Boeing, W.J. (2010) Low genetic differentiation among populations of the great plains toad (*Bufo cognatus*) in Southern New Mexico. *Copeia*, 388-396.

Korn, M., Green, A.J., Machado, M., García-de-Lomas, J., Cristo, M., da Fonseca, L.C., Frisch, D., Pérez-Bote, J.L. & Hundsdoerfer, A.K. (2010) Phylogeny, molecular ecology and taxonomy of southern Iberian lineages of *Triops mauritanicus* (Crustacea: Notostraca). *Organisms Diversity & Evolution*, **10**, 409-440.

Loftis, D.G., Echelle, A.A., Koike, H., Van Den Bussche, R.A. & Minckley, C.O. (2009) Genetic structure of wild populations of the endangered Desert Pupfish complex (Cyprinodontidae: Cyprinodon). *Conservation Genetics*, **10**, 453-463.

Lopes-Cunha, M., Aboim, M.A., Mesquita, N., Alves, M.J., Doadrio, I. & Coelho, M. (2012) Population genetic structure in the Iberian cyprinid fish *Iberochondrostoma lemmingii* (Steindachner, 1866): disentangling species fragmentation and colonization processes. *Biological Journal of the Linnean Society*, **105**, 559-572.

Mamuris, Z., Stoumboudi, M.T., Stamatis, C., Barbieri, R. & Moutou, K.A. (2005) Genetic variation in populations of the endangered fish *Ladigesocypris ghigii* and its implications for conservation. *Freshwater biology*, **50**, 1441-1453.

Martin, A.P. (2010) The conservation genetics of Ash Meadows pupfish populations. I. The Warm Springs pupfish *Cyprinodon nevadensis pectoralis*. *Conservation Genetics*, **11**, 1847-1857.

Martin, A.P. & Wilcox, J.L. (2004) Evolutionary history of Ash Meadows pupfish (genus *Cyprinodon*) populations inferred using microsatellite markers. *Conservation Genetics*, **5**, 769-782.

Masci, K.D., Ponniah, M. & Hughes, J.M. (2008) Patterns of connectivity between the Lake Eyre and Gulf drainages, Australia: a phylogeographic approach. *Marine and Freshwater Research*, **59**, 751-760.

McGaugh, S.E. (2012) Comparative population genetics of aquatic turtles in the desert. *Conservation Genetics*, **13**, 1561-1576.

Mesquita, N., Hanfling, B., Carvalho, G.R. & Coelho, M.M. (2005) Phylogeography of the cyprinid *Squalius aradensis* and implications for conservation of the endemic freshwater fauna of southern Portugal. *Molecular Ecology*, **14**, 1939-1954.

Mesquita, N., Carvalho, G., Shaw, P., Crespo, E. & Coelho, M.M. (2001) River basin‐related genetic structuring in an endangered fish species, *Chondrostoma lusitanicum*, based on mtDNA sequencing and RFLP analysis. *Heredity*, **86**, 253-264.

Miller, M.P., Blinn, D.W. & Keim, P. (2002) Correlations between observed dispersal capabilities and patterns of genetic differentiation in populations of four aquatic insect species from the Arizona White Mountains, USA. *Freshwater Biology*, **47**, 1660-1673.

Miller, M.P., Weigel, D.E. & Mock, K.E. (2006) Patterns of genetic structure in the endangered aquatic gastropod *Valvata utahensis* (Mollusca: Valvatidae) at small and large spatial scales. *Freshwater Biology*, **51**, 2362-2375.

Miller, M.P., Stevens, L.E., Busch, J.D., Sorensen, J.A. & Keim, P. (2000) Amplified fragment length polymorphism and mitochondrial sequence data detect genetic differentiation and relationships in endangered southwestern USA ambersnails (*Oxyloma* spp.). *Canadian Journal of Zoology*, **78**, 1845-1854.

Mock, K.E. & Miller, M. (2005) Patterns of molecular diversity in naturally occurring and refugial populations of the least chub. *Transactions of the American Fisheries Society*, **134**, 267-278.

Mock, K.E., Brim‐Box, J., Miller, M., Downing, M. & Hoeh, W. (2004) Genetic diversity and divergence among freshwater mussel (*Anodonta*) populations in the Bonneville Basin of Utah. *Molecular Ecology*, **13**, 1085-1098.

Mock, K.E., Evans, R., Crawford, M., Cardall, B., Janecke, S.U. & Miller, M. (2006) Rangewide molecular structuring in the Utah sucker (*Catostomus ardens*). *Molecular Ecology*, **15**, 2223-2238.

Mock, K.E., Brim Box, J.C., Chong, J.P., Howard, J.K., Nez, D.A., Wolf, D. & Gardner, R.S. (2010) Genetic structuring in the freshwater mussel *Anodonta* corresponds with major hydrologic basins in the western United States. *Molecular Ecology*, **19**, 569-591.

Moline, A.B., Shuster, S.M., Hendrickson, D.A. & Marks, J.C. (2004) Genetic variation in a desert aquatic snail (*Nymphophilus minckleyi*) from Cuatro Cienegas, Coahuila, Mexico. *Hydrobiologia*, **522**, 179-192.

Morales, P., Vila, I. & Poulin, E. (2011) Genetic structure in remnant populations of an endangered cyprinodontid fish, *Orestias ascotanensis*, endemic to the Ascotán salt pan of the Altiplano. *Conservation Genetics*, **12**, 1639-1643.

MuNOz, J., GOMez, A., Green, A.J., Figuerola, J., Amat, F. & Rico, C. (2008) Phylogeography and local endemism of the native Mediterranean brine shrimp *Artemia salina* (Branchiopoda: Anostraca). *Molecular Ecology*, **17**, 3160-3177.

Murphy, N.P. & Austin, C.M. (2004) Phylogeography of the widespread Australian freshwater prawn, *Macrobrachium australiense* (Decapoda, Palaemonidae). *Journal of Biogeography*, **31**, 1065-1072.

Murphy, N.P., Adams, M. & Austin, A.D. (2009) Independent colonization and extensive cryptic speciation of freshwater amphipods in the isolated groundwater springs of Australia's Great Artesian Basin. *Molecular Ecology*, **18**, 109-122.

Murphy, N.P., Guzik, M.T. & Wilmer, J.W. (2010) The influence of landscape on population structure of four invertebrates in groundwater springs. *Freshwater Biology*, **55**, 2499-2509.

Murphy, N.P., Adams, M., Guzik, M.T. & Austin, A.D. (2013) Extraordinary micro-endemism in Australian desert spring amphipods. *Molecular Phylogenetics and Evolution*, **66**, 645-653.

Murphy, N.P., Breed, M.F., Guzik, M.T., Cooper, S.J.B. & Austin, A.D. (2012) Trapped in desert springs: phylogeography of Australian desert spring snails. *Journal of Biogeography*,

Nguema, R.M., Langand, J., Galinier, R., Idris, M.A., Shaban, M.A., Al Yafae, S., Moné, H. & Mouahid, G. (2013) Genetic diversity, fixation and differentiation of the freshwater snail *Biomphalaria pfeifferi* (Gastropoda, Planorbidae) in arid lands. *Genetica*, **141**, 171-184.

Nguyen, T.T.T., Austin, C.M., Meewan, M.M., Schultz, M.B. & Jerry, D.R. (2004) Phylogeography of the freshwater crayfish *Cherax destructor* Clark (Parastacidae) in inland Australia: historical fragmentation and recent range expansion. *Biological Journal of the Linnean Society*, **83**, 539-550.

Nielsen, J.L. & Sage, G.K. (2002) Population genetic structure in Lahontan cutthroat trout. *Transactions of the American Fisheries Society*, **131**, 376-388.

Pamponet, V.D.C., Carneiro, P.L.S., Affonso, P., Miranda, V.S., Silva, J.C., de Oliveira, C.G. & Gaiotto, F.A. (2008) A multi-approach analysis of the genetic diversity in populations of *Astyanax* aff. *bimaculatus* Linnaeus, 1758 (Teleostei: Characidae) from Northeastern Brazil. *Neotropical Ichthyology*, **6**, 621-630.

Phillipsen, I.C. & Metcalf, A.E. (2009) Phylogeography of a stream-dwelling frog (*Pseudacris cadaverina*) in southern California. *Molecular Phylogenetics and Evolution*, **53**, 152-170.

Phillipsen, I.C., Kirk, E.H., Bogan, M.T., Mims, M.C., Olden, J.D. & Lytle, D.A. (2015) Dispersal ability and habitat requirements determine landscape‐level genetic patterns in desert aquatic insects. *Molecular Ecology*, **24**, 54-69.

Ponder, W.F., Eggler, P. & Colgan, D.J. (1995) Genetic differentiation of aquatic snails (Gastropoda: Hydrobiidae) from artesian springs in arid Australia. *Biological Journal of the Linnean Society*, **56**, 553-596.

Pritchard, V., Metcalf, J., Jones, K., Martin, A. & Cowley, D. (2009) Population structure and genetic management of Rio Grande cutthroat trout (*Oncorhynchus clarkii virginalis*). *Conservation Genetics*, **10**, 1209-1221.

Quattro, J.M., Leberg, P.L., Douglas, M.E. & Vrijenhoek, R.C. (1996) Molecular evidence for a unique evolutionary lineage of endangered Sonoran desert fish (Genus *Poeciliopsis*). *Conservation Biology*, **10**, 128-135.

Robertson, H.L., Guzik, M.T. & Murphy, N.P. (2014) Persistence in the desert: ephemeral waterways and small-scale gene flow in the desert spring amphipod, *Wangiannachiltonia guzikae*. *Freshwater Biology*, **59**, 653-665.

Schwentner, M., Timms, B.V. & Richter, S. (2012) Flying with the birds? Recent large-area dispersal of four Australian *Limnadopsis* species (Crustacea: Branchiopoda: Spinicaudata). *Ecology and Evolution*, **2**, 1605-1626.

Schwentner, M., Timms, B.V. & Richter, S. (2014) Evolutionary systematics of the Australian Eocyzicus fauna (Crustacea: Branchiopoda: Spinicaudata) reveals hidden diversity and phylogeographic structure. *Journal of Zoological Systematics and Evolutionary Research*, **52**, 15-31.

Sei, M., Lang, B.K. & Berg, D.J. (2009) Genetic and community similarities are correlated in endemic-rich springs of the northern Chihuahuan Desert. *Global Ecology and Biogeography*, **18**, 192-201.

Seidel, R.A., Lang, B.K. & Berg, D.J. (2009) Phylogeographic analysis reveals multiple cryptic species of amphipods (Crustacea: Amphipoda) in Chihuahuan Desert springs. *Biological Conservation*, **142**, 2303-2313.

Small, M.P., Burgess, D., Dean, C. & Warheit, K.I. (2011) Does Lower Crab Creek in the Eastern Washington Desert Have a Native Population of Chinook Salmon? *Transactions of the American Fisheries Society*, **140**, 808-821.

Sousa-Santos, C., Robalo, J.I., Francisco, S.M., Carrapato, C., Cardoso, A.C. & Doadrio, I. (2014a) Metapopulations in temporary streams–The role of drought–flood cycles in promoting high genetic diversity in a critically endangered freshwater fish and its consequences for the future. *Molecular Phylogenetics and Evolution*, **80**, 281-296.

Sousa-Santos, C., Gante, H.F., Robalo, J., Cunha, P.P., Martins, A., Arruda, M., Alves, M. & Almada, V. (2014b) Evolutionary history and population genetics of a cyprinid fish (*Iberochondrostoma olisiponensis*) endangered by introgression from a more abundant relative. *Conservation Genetics*, **15**, 665-677.

Sousa, V., Penha, F., Collares-Pereira, M.J., Chikhi, L. & Coelho, M.M. (2008) Genetic structure and signature of population decrease in the critically endangered freshwater cyprinid *Chondrostoma lusitanicum*. *Conservation Genetics*, **9**, 791-805.

Sousa, V., Penha, F., Pala, I., Chikhi, L. & Coelho, M.M. (2010) Conservation genetics of a critically endangered Iberian minnow: evidence of population decline and extirpations. *Animal Conservation*, **13**, 162-171.

Stutz, H.L., Shiozawa, D.K. & Evans, R.P. (2010) Inferring dispersal of aquatic invertebrates from genetic variation: a comparative study of an amphipod and mayfly in Great Basin springs. *Journal of the North American Benthological Society*, **29**, 1132-1147.

Thomas, P.E., Blinn, D.W. & Keim, P. (1997) Genetic and behavioural divergence among desert spring amphipod populations. *Freshwater Biology*, **38**, 137-143.

Tibbets, C.A. & Dowling, T.E. (1996) Effects of intrinsic and extrinsic factors on population fragmentation in three species of North American minnows (Teleostei: Cyprinidae). *Evolution*, 1280-1292.

Van Leeuwen, C.H.A., Huig, N., Van der Velde, G., Van Alen, T.A., Wagemaker, C.A.M., Sherman, C.D.H., Klaassen, M. & Figuerola, J. (2013) How did this snail get here? Several dispersal vectors inferred for an aquatic invasive species. *Freshwater Biology*, **58**, 88-99.

Velo-Antón, G., Godinho, R., Campos, J.C. & Brito, J.C. (2014) Should I Stay or Should I Go? Dispersal and population structure in small, isolated desert populations of West African Crocodiles. *PloS One*, **9**, e94626.

Viard, F., Justy, F. & Jarne, P. (1997) The influence of self-fertilization and population dynamics on the genetic structure of subdivided populations: a case study using microsatellite markers in the freshwater snail *Bulinus truncatus*. *Evolution*, 1518-1528.

Wang, I.J. (2009) Fine-scale population structure in a desert amphibian: landscape genetics of the black toad (*Bufo exsul*). *Molecular Ecology*, **18**, 3847-3856.

Whitehead, A., Anderson, S.L., Kuivila, K.M., L Roach, J. & May, B. (2003) Genetic variation among interconnected populations of *Catostomus occidentalis*: implications for distinguishing impacts of contaminants from biogeographical structuring. *Molecular Ecology*, **12**, 2817-2833.

Woods, R.J., Macdonald, J.I., Crook, D.A., Schmidt, D.J. & Hughes, J.M. (2010) Contemporary and historical patterns of connectivity among populations of an inland river fish species inferred from genetics and otolith chemistry. *Canadian Journal of Fisheries and Aquatic Sciences*, **67**, 1098-1115.

Worthington Wilmer, J., Elkin, C., Wilcox, C., Murray, L., Niejalke, D. & Possingham, H. (2008) The influence of multiple dispersal mechanisms and landscape structure on population clustering and connectivity in fragmented artesian spring snail populations. *Molecular Ecology*, **17**, 3733-3751.

Zickovich, J.M. & Bohonak, A.J. (2007) Dispersal ability and genetic structure in aquatic invertebrates: a comparative study in southern California streams and reservoirs. *Freshwater Biology*, **52**, 1982-1996.

**Appendix S2**. Proportion of studies of desert freshwater taxa that concluded three categories of connectivity, at three different scales, compared between the three most-studied taxonomic classes.
